# Interventional effects of the direct application of “Sanse powder” on knee osteoarthritis in rats as determined from lipidomics via UPLC-Q-Exactive Orbitrap MS

**DOI:** 10.1186/s13020-020-0290-5

**Published:** 2020-01-23

**Authors:** Peng Wu, Zhengquan Huang, Jinjun Shan, Zichen Luo, Nongshan Zhang, Songjiang Yin, Cunsi Shen, Runlin Xing, Wei Mei, Yancheng Xiao, Bo Xu, Jun Mao, Peimin Wang

**Affiliations:** 1 Affiliated Hospital of Nanjing University of Chinese Medicine, Nanjing, 210029 China; 2 Jiangsu Province Hospital of Chinese Medicine, Nanjing, 210029 China; 3 Medical Metabolomics Center, Nanjing University of Chinese Medicine, Nanjing, 210023 China; 4 Jiangsu Key Laboratory of Pediatric Respiratory Disease, Institute of Pediatrics, Nanjing University of Chinese Medicine, Nanjing, 210023 China; 5 Key Laboratory for Metabolic Diseases in Chinese Medicine, First College of Clinical Medicine, Nanjing University of Chinese Medicine, Nanjing, 210023 China

**Keywords:** KOA lipidomics, LC–MS, Direct application “Sanse powder” therapy, Prescription

## Abstract

**Background:**

Our previous clinical evidence suggested that the direct application of “Sanse powder” the main ingredient of “Yiceng” might represent an alternative treatment for knee osteoarthritis. However, the mechanism underlying its effect is poorly understood. In this study, we investigated the mechanism of the effect of direct “Sanse powder” application for the treatment of knee osteoarthritis (KOA) in rats by using lipidomics.

**Methods:**

KOA rats were established by cutting the anterior cruciate ligament, and the cold pain threshold and mechanical withdrawal threshold (MWT) of seven rats from each group were measured before modelling (0 days) and at 7, 14, 21 and 28 days after modelling. Histopathological evaluation of the synovial tissue was performed by haematoxylin and eosin (H&E) staining after modelling for 28 days. Interleukin-1β (IL-1β), pro-interleukin-1β (pro-IL-1β) and tumor necrosis factor-α (TNF-α) proteins in synovial tissue were measured by western blot, and the mRNA expression levels of IL-1β and TNF-α in synovial tissue were measured using Real-time reverse transcription polymerase chain reaction (qRT-PCR), the levels of IL-1β and TNF-α in rat serum were measured by enzyme-linked immunosorbent assay (ELISA), Serum lipid profiles were obtained by using ultra-performance liquid chromatography combined with quadrupole-Exactive Orbitrap mass spectrometry (UPLC-Q-Exactive Orbitrap MS).

**Results:**

The results confirmed that the direct application of “Sanse powder” had a significant protective effect against KOA in rats. Treatment with “Sanse powder” not only attenuated synovial tissue inflammation but also increased the levels of the cold pain threshold and MWT. In addition, the lipidomics results showed that the levels of diacylglycerol (DAG), triacylglycerols (TAGs), lysophosphatidylcholine (LPC), phosphatidylcholine (PC), fatty acid esters of hydroxy fatty acids (FAHFAs), and phosphatidylethanolamine (PE) were restored almost to control levels following treatment.

**Conclusions:**

Lipidomics provides a better understanding of the actions of direct application “Sanse powder” therapy for KOA.

## Background

Increasing research suggests that knee osteoarthritis (KOA) is a total joint disease involving cartilage, subchondral bone, ligaments, the meniscus and the synovium [1]. Synovial inflammation may be important in the pathogenesis of KOA and is associated with pain. To date, there is no specific treatment for KOA [2]. Pharmacological therapy may include the paracetamol, non-steroidal anti-inflammatory drugs (NSAIDs) or intra-articular corticosteroids, However, the long-term use of NSAIDs can lead to adverse reactions, such as peptic ulcers and bleeding, and although selective COX-2 inhibitors can effectively reduce the incidence of similar events, they can have adverse effects on the cardiovascular system [3–5].

Many studies have shown significant treatment effects of traditional Chinese medicine on KOA. “Sanse powder” is the main component of “Yiceng”, and our previous studies found that “Yiceng” application alleviated synovial inflammation, relieved pain and improved the cold pain threshold [6, 7]. However, the mechanisms underlying these effects remain unclear.

The close relationship between lipid metabolism and KOA has attracted widespread attention. Lipid metabolism imbalance can induce KOA, and KOA can also lead to lipid metabolism disorders. Lipidomics is increasingly recognized as an invaluable tool for identifying changes in lipid metabolites and reveal the underlying mechanisms [8, 9]. As there are many kinds of lipids and as the substrates of biological samples are complex, so the analysis of lipidomics requires advanced separation technology and detection. Liquid chromatography-tandem mass spectrometry (LC-MS) is effective for the analysis of lipids in biological samples. Ultra-performance liquid chromatography (UPLC) enables the rapid and effective separation of individual lipid species. Lipidomics based on UPLC combined with quadrupole-Exactive Orbitrap mass spectrometry (UPLC-Q-Exactive MS) has been widely applied to obtain insight into many biological events [10]. In this study, UPLC-Q-Exactive MS was used to analyse various lipids in the serum of KOA rats treated with “Sanse powder”. An experiment was conducted to evaluate the effects of adhesive “Sanse powder” treatment on the lipid profile. The results provide a new understanding of the effects of this treatment for KOA.

## Methods

### Preparation of “Sanse powder” patches

“Sanse powder” patches were created from eleven Chinese material medica (CMMS), *Forsythia suspensa*, Glycyrrhiza uralensis, Salvia miltiorrhiza, *Gentiana macrophylla*, Chaenomeles sinensis, *Strychnos nux-vomica*, Ligusticum striatum Hort, *Curcuma longa*, Paeonia lactiflora, Notopterygium root and Saposhnikovia divaricata, all at equal ratios. All of the CMMs were purchased from Anhui Wansheng Traditional Chinese Medicine Decoction Pieces Co., Ltd., (Anhui, China) and authenticated by Professor Peimin Wang from Affiliated Hospital of Nanjing University of Chinese Medicine (Nanjing, China). All the herbal materials used in our study satisfy the quality requirements of the 2015 edition of the Chinese Pharmacopoeia. All the herbs were ground into powder and mixed with Vaseline at a 1:1 ratio to create the patches.

### Animals

Twenty-one male Sprague–Dawley rats weighting 150 g to 180 g were purchased from Nanjing Qinglongshan Animal Farm (animal certificate number: SCXK-Zhe-2014-0001). All animal procedures were performed in accordance with the Guidelines for Care and Use of Laboratory Animals of Nanjing University of Chinese Medicine. The rats were housed in an environment with a room temperature of 24–26 °C, a relative humidity of 55%, and a 12-h light–dark cycle. The experiments were approved by the Animal Ethics Committee of Nanjing University of Chinese Medicine. The project identification code of the ethical statement is 201810A001 (Additional file 1).

One week after adaptive feeding, the rats were randomly grouped into three groups: a control group (n = 7), a KOA group (n = 7), and a “Sanse powder” group (KOA+Sanse powder, n = 7). The KOA model was constructed by anterior cruciate ligament transection (ACLT) surgery, as described previously, on both knees [11]. The drug was administered to the “Sanse powder” group after preparation of the KOA model. The knees were skinned and “Sanse powder” paste (0.8 g/10 cm^2^) was evenly applied to the skin surface for 8 h a day for a total of 14 days. After the 14 days, the rats were sacrificed to harvest the cartilage, synovial tissue and plasma.

### Sample collection

After the final treatment administration, the rats were anaesthetized by intraperitoneal injection with 3% pentobarbital sodium and sacrificed after sampling blood from the abdominal aorta (CO_2_ asphyxiation). The knees were skinned close to the patellar ligament on both sides of the cut and the superior border of the patella. A distal lateral incision in the quadriceps was made to the femur, and the patella and its surrounding tissues were obtained with ophthalmic forceps. The surgical blade was manipulated carefully to avoid damaging the transparent synovial tissue. The synovial tissue from two randomly selected rats from each group was preserved in 4% paraformaldehyde for pathological section, and the remaining the synovial tissues from the groups were cryopreserved at − 80 °C.

### Histopathology

After 2 weeks, cartilage was taken from the control and model KOA groups, fixed in 10% neutral formalin, embedded in paraffin cut into slices, and stained by haematoxylin and eosin (H&E) staining. After 14 days of administration, rats were sacrificed to harvest the synovial tissue and serum, and the synovial tissue was treated as cartilage.

### Measurements of inflammatory mediators

#### Western blotting

Western blotting was performed as previously described [12]. The blots were incubated with primary antibodies, including anti-IL-1β (1:1000, Abcam, Cambridge, UK), anti-pro-IL-1β (1:1000, Abcam, Cambridge, UK) and anti-TNF-α (1:1000, Abcam, Cambridge, UK).

#### Real-time reverse transcription polymerase chain reaction (qRT-PCR)

qRT-PCR was performed as previously described [12]. Primer was designed and synthesized by Shanghai Biotechnology Service Company in accordance with Gene sequence in GenBank Gene sequence design, together with Oligo v6.6. Sequences for primes were as follows: TNF-α forward, 5’-TGGGCTCCCTCTCATCAGTTC-3’ and reverse, 5’-GCTCCTCCGCTTGGTGGTTTG-3’; IL-1β forward, 5’-ACAGCAGCATCTCGACAAGAGC-3’ and reverse, 5’-CCACGGGCAAGACATAGGTAGC-3’; GAPDH forward, 5’-GATGCTGGTGCTGAGTATG-3’ and reverse, 5’-GTGGTGCAGGATGCATTGCT-3’.

#### Enzyme-linked immunosorbent assay (ELISA)

The leves of IL-1β and TNF-α in rat serum were estimated using a rat ELISA kit (FcMACS, Nanjing, China) according to manufacturer instructions.

### Measurements of the cold pain threshold and mechanical withdrawal threshold (MWT)

#### Cold pain threshold

Cold pain sensitivity was determined at 0, 7, 14, 21, and 28 days after modelling, The measurement was performed at 7:00 AM by placing a rat on a cold plate (35150-001, Ugo Basil SLR, Italy) with a temperature of 0 ± 3 °C [13], covering the rats with an organic cylinder and recording the time from contact with the cold plate to the time of rat rapid paw withdrawal, paw licking, foot stamping or jumping; paw withdrawal due to physical activity was not considered a positive reaction. The measurement was performed 3 times with 10 min between each test, and the average value was obtained.

#### MWT

The MWT was measured at 0, 7, 14, 21, and 28 days after modelling. Measurements were taken at 14:00, and all rats were acclimated to the testing environment 5 min before the test. The rats were placed in Plexiglass cages with wire mesh (BME-404, Institute of Biomedical Engineering, Chinese Academy of Medical Sciences). An electronic needle was used to stimulate the sole of the right hind foot. The stimulation intensity was increased gradually with the needle gradually bending until the rats exhibited rapid paw withdrawal, lifting, and licking reactions within 3 s. A computer automatically displayed the minimal force on the needle to induce paw withdrawal. The measurement was made 3 times, with 10 min between each test, and the average value was obtained.

### Processing and determination of “Sanse powder”

One gram of “Sanse powder” was placed in a conical flask with 25 mL methanol. After 45 min of ultrasonic oscillation, the extract was centrifuged at 18,000 rpm, and the supernatant was used for mass spectrometric analysis. The following standards were used: curcumin (32.24 μg/mL), ligustilide (32.63 μg/mL), tanshinone I (28.03 μg/mL), imperatorin (32.89 μg/mL), gentiopicroside (34.34 μg/mL), osthole (35.66 μg/mL), oleanolic acid (31.18 μg/mL), quercetin (35.13 μg/mL), and paeoniflorin (33.42 μg/mL). The standards were mixed together to create a standard solution, and the fragment ions and retention time were compared with those of samples by chromatographic and mass spectrometric analysis.

A UPLC instrument (Waters, USA) was used to separate the samples. Acetonitrile (A) and 0.1% aqueous formic acid (B) were selected as the mobile phases. The gradient mobile phase programme was as follows: 0 min, 10% A; 50 min, 90% A; 52 min, 90% A; 55 min, 10% A; and 60 min, 10% A. The column oven temperature was 25 °C, and the flow rate was 0.4 mL/min. A Hypersil GOLD column (100 × 2.1 mm, Thermo, USA) was used.

An LTQ-Orbitrap XL mass spectrometer was used to analyse the samples, and the detection conditions were the same in both positive and negative ion modes. The sheath gas flow rate and aux gas flow rate were 45 arb and 10 arb, respectively. The heater temperature and capillary temperature were both 300 °C, and the capillary voltage was 35 V.

### Analysis conditions of UPLC-Q-Exactive MS

#### Treatment of the serum samples of KOA rats

Serum was thawed to 4 °C, and 20 μL of serum was placed into a 1.5 mL centrifuge tube, to which 225 μL of cold methanol was added. The internal standard (lyso-PE (17:1), SM (17:0), Avanti Polar Lipids, United States, approximate concentration: 5 μg/mL) was also added. The mixture was vortexed (Scientific Industries, USA) for 10 s, and 750 μL MTBE was added. The mixture was vortexed for 10 s and then oscillated for 10 min at 4 °C. Ultrapure water (188 μL) was then added, and the mixture was vortexed for 20 s and then centrifuged at 18,000 rpm Beckman, USA for 2 min at 4 °C. A total of 350 μL of the supernatant solution was placed into a 1.5 mL centrifuge tube, which was placed in a centrifuge concentrator (Thermo, USA). The samples were redissolved in 110 μL of methanol: toluene (9:1), vortexed for 10 min, and sonicated (Kunshan Ultrasonic Instrument Co., Ltd.). The samples were then centrifuged for 10 min at 18,000 rpm, and samples of the supernatant were collected for analysis.

The QC samples were prepared by combining 10 μL samples in the same centrifuge tube, and 20 μL of sample were then collected and treated according to the above experimental steps.

#### Chromatographic conditions

A U3000 high-performance liquid chromatograph (Dionex, USA) was used to separate the samples and was equipped with an ACQUITY CSH C18 column (1.7 μm, 2.1 × 100 mm). The flow rate was 0.3 mL/min, and the mobile phase in positive ion mode was (A) 6:4 acetonitrile: water + 10 mM ammonium formate + 0.1% formic acid and (B) 9:1 isopropanol: acetonitrile + 10 mM ammonium formate + 0.1% formic acid. The mobile phase in negative mode was (A) 6:4 acetonitrile: water + 10 mM ammonium acetate and (B) 9:1 isopropanol: acetonitrile + 10 mM ammonium acetate. The gradient of the mobile phase was as follows: 0–2 min, 15–30% B; 2–2.5 min, 30–48% B; 2.5–11 min, 48–82% B; 11–11.5 min, 82–99% B; 11.5–12 min, 99% B; 12–12.1 min, 99–15% B; and 12.1–15 min, 15% B. The column temperature was 65 °C, and the injection volume was 2 μL in both positive and negative ion mode.

#### Mass spectrometric conditions

A Q-Exactive four-stage pole-orbit well mass spectrometer (Thermo Fisher Scientific) was used to detect samples. A hybrid quadrupole-Exactive Orbitrap mass spectrometer was coupled to an HESI source. The instrument parameters for position ion mode were as follows: spray voltage, 3.5 kV; heater temperature, 306 °C; capillary temperature, 300 °C; sheath gas flow, 45 arb; aux gas flow, 10 arb; scanning range, 215–1800 m/z; and s-lens, 50. The parameters for negative mode were as follows: spray voltage, 3.0 kV; heater temperature, 325 °C; capillary temperature, 300 °C; sheath gas flow, 45 arb; aux gas flow, 10 arb; scanning range, 215–1800 m/z; and s-lens, 50.

### Data processing method

Using the software Abf Converter (http://www.reifycs.com/AbfConverter/), the raw files were converted into Abf format, and the positive and negative ion mode data were imported into MS-DIAL software for data preprocessing, filtering, alignment and peak identification. Related software parameters are listed in the following table. The identified metabolites, retention times (Rt), mass-to-charge ratios (m/z) and high peak value information were obtained for all samples. The data exported from MS-DIAL were normalized by the SERRF algorithm through the R language. The R language was used to compare FCs with the median peak height of each group. An FC > 1.5 was considered to indicate a significantly difference. Non-parametric analysis and FDR analysis were used to analyse the differences between groups and obtain *P* values. An FDR-adjusted *P *< 0.05 was considered to indicate significance. Using Metaboanalyst (http://www.metaboanalyst.ca/), data on principal component analysis and cluster analysis were conducted.

## Results

### “Sanse powder” can alleviate the symptoms of KOA

**Fig. 1:**
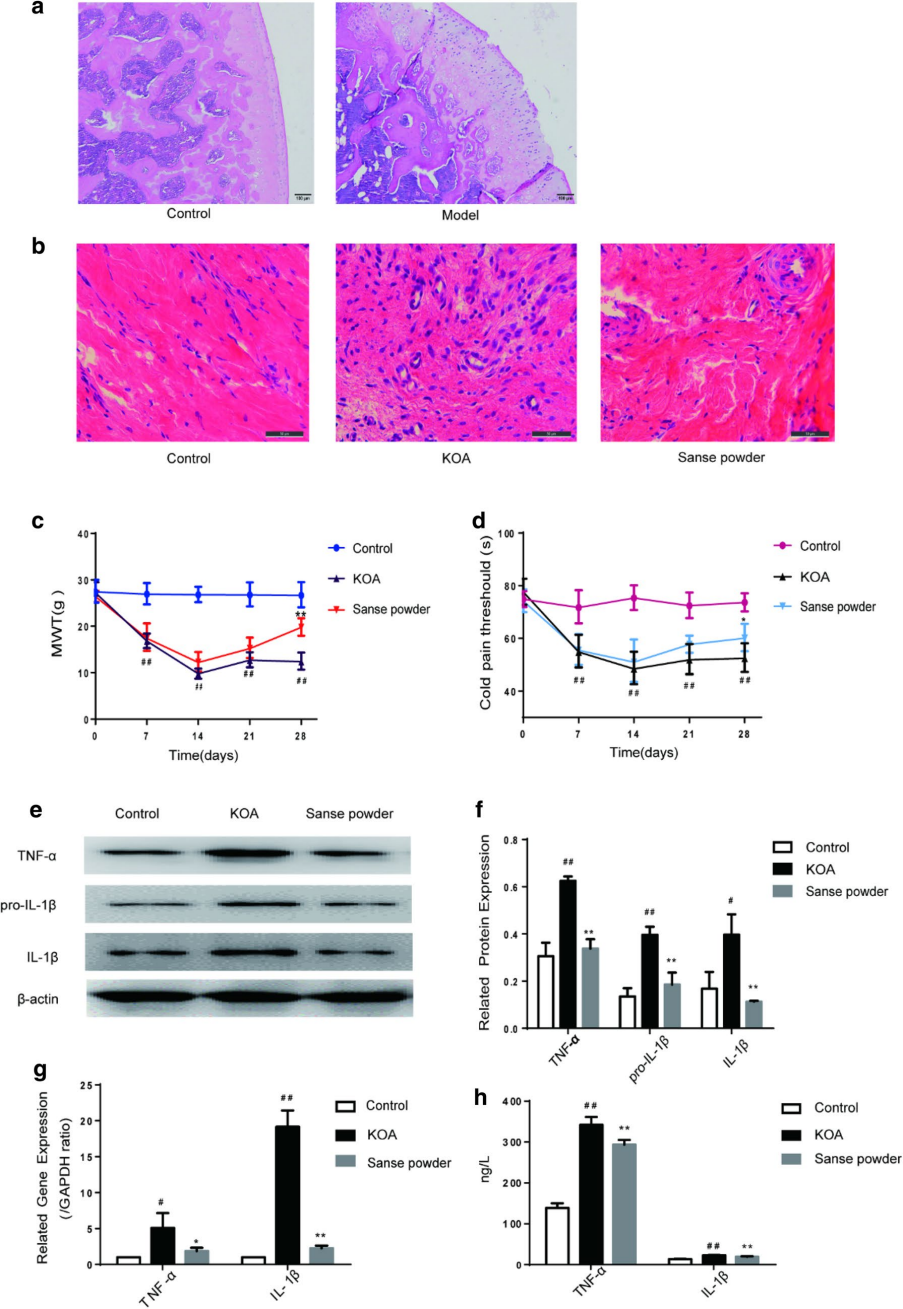
“Sanse powder” can alleviate the symptoms of KOA. Notes. **a** Cartilage histomorphology of each group stained with H&E, 40×, scale bar = 100 μm. **b** Representative synovial tissues of each group stained with H&E, 400×, scale bar = 50 um. **c**, **d** MWT and cold pain threshold of each group. ^#^*P *< 0.05, ^##^*P *< 0.01 vs. control group; *P < 0.05, **P < 0.01 vs KOA group (n = 7). **e**, **f** Representative protein bands for each group. ^#^*P* < 0.05, ^##^*P* < 0.01 vs. control group; *P < 0.05, **P < 0.01 vs. KOA group. **g** Representative gene bands for each group. ^#^*P* < 0.05, ^##^*P* < 0.01 vs. control group; *P < 0.05, **P < 0.01 vs. KOA group. **h** Levels of TNF-α and IL-1β in rat serum were detected by ELISA. ^#^*P* < 0.05, ^##^*P* < 0.01 vs. control group; *P < 0.05, **P < 0.01 vs. KOA group

After 14 days of modelling, 1 rat from each of the control group and the model group was selected randomly. Histopathology analysis of the cartilage of rats (Fig. 1a) in the control and model groups was performed to confirm that the KOA model had been successfully established. In the control group, the cartilage surface was smooth with no cracks or defects, and the chondrocytes were arranged in an orderly manner, with normal structural layering and uniform staining. In the model group, the cartilage surface was severely altered and had many cracks, with some extending down to the radiation layer, and exhibited unclear stratification, an irregular structure of each layer and pronounced thickening of the calcification layer. In the HE staining section of the synovial membrane, the cells in the control group were arranged in an orderly manner, with almost no inflammatory cell infiltration. In the model group, inflammatory cell infiltration was significantly increased, the synovial cells had proliferated to large numbers and were irregularly arranged, and defects were apparent around the synovial tissue, presenting pathological changes of severe synovitis. The infiltration of inflammatory cells in the “Sanse powder” group was significantly reduced related to that in the KOA group (Fig. 1b). We also observed that “Sanse powder” treatment increased the cold pain threshold and MWT in KOA rats (Fig. 1c, d). We also analysed the relative protein and gene expressions in synovial tissue and the levels of TNF-α and IL-1β in the rat serum. The results showed that the “Sanse powder” group exhibited significant downregulation of those proteins relative to the expression in the KOA group (Fig. 1e–h).

### Chemical constituents of “Sanse powder” prescription

**Fig. 2:**
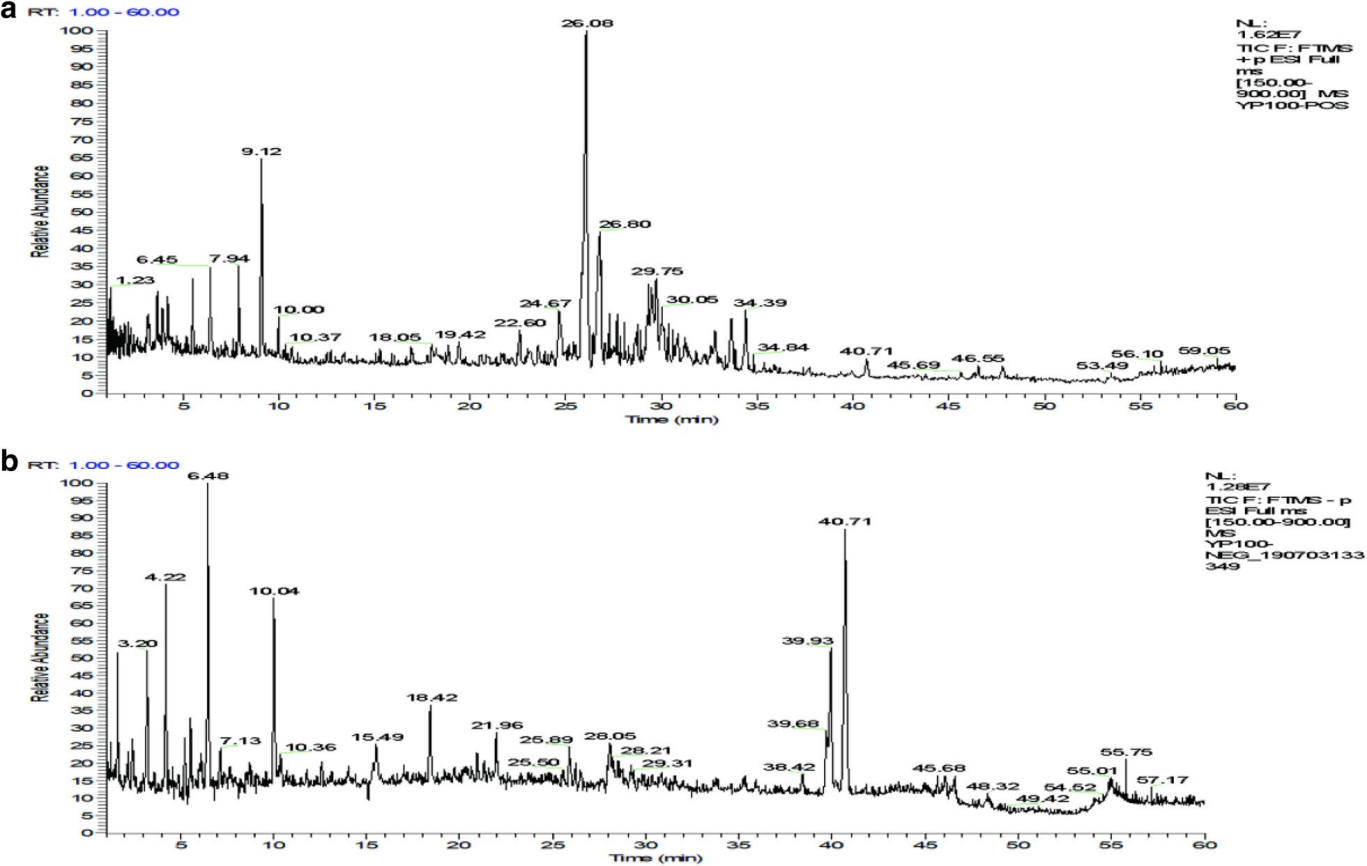
Total ion chromatogram (TIC) of the methanol extract acquired by LTQ-Orbitrap MS in the positive (**a**) and negative (**b**) ion modes

By comparing the accurate m/z and mass fragmentation pattern with those in the literatures, thirty-five components were tentatively identified by LTQ-Orbitrap MS, including curcumin, ligustilide, and tanshinone I. The base peak chromatogram of the extract of “Sanse powder” is depicted in Fig. 2, and a summary of lipid metabolites detected by LTQ-Orbitrap MS is shown in Table 1. The compounds detected were divided into several categories, including alkaloids, saponins, lactones, and organic acids. Six ingredients from Forsythia suspensa were identified. Three ingredients were from Glycyrrhiza uralensis, six ingredients were from Salvia miltiorrhiza, four ingredients were from Gentiana macrophylla, three ingredients were from Chaenomeles sinensis, four ingredients were from Strychnos nux-vomica, five ingredients were from Ligusticum striatum Hort, one ingredient was from Curcuma longa, one ingredient was from Paeonia lactiflora, one ingredient was from Notopterygium root and one ingredients was from Saposhnikovia divaricata. Most of the ingredients have been reported possess anti-inflammatory and analgesic activity. For example, gentiopicroside exerted anti-inflammatory effects in vivo by inhibiting prostaglandin E2 (PGE2) release and type II collagen synthesis in the presence of IL-1β through the MAPK signaling pathways that prevent P38, ERK, and JNK phosphorylation [14]. Phillyrin, an active ingredient extracted from forsythia, significantly inhibited receptor activator of nuclear factor κB ligand (RANKL)-induced osteoclastogenesis and bone resorption in vitro and protected against lipopolysaccharide-induced osteolysis in vivo. Phillyrin effectively blocked RANKL-induced activations of c-Jun N-terminal kinase and extracellular signal-regulated kinase, which suppressed the expression of c-Fos and nuclear factor of activated T-cells cytoplasmic 1 [15]. Imperatorin (IMT) reduced the release of TNF–α, IL-6 and IL-1β, inhibited the expression of iNOS and COX-2, and suppressed the activity of NF-κB via upregulation of the expression of p65 (C) and IκB (C), and downregulation of the expression of p65 (N) [16]. Quercetin alleviated rat osteoarthritis by inhibiting inflammation and apoptosis of chondrocytes, modulating synovial macrophages polarization to M2 macrophages [17]. The pharmacological effects of Sanse powder may be a synergy of actions of multicomponents. Further studies are needed to elucidate the protective molecular mechanisms of Sanse powder on KOA in detail.

**Table 1:** The chemical components identified in the “Sanse powder” water extract

(A) positive ion mode					
No.	Components	Chemical formula	ESI^+^, m/z	RT (min)	References
1	Arginine	C_6_H_14_N_4_O_2_	175.11895 [M+H]^+^, 116.70, 175.19, 71.84	0.63	[21]
2	Gentiopicroside	C_16_H_20_O_9_	357.11800	3.20	*
3	Ferulic acid	C_10_H_10_O_4_	195.06519 [M+H]^+^, 176.91, 137.88, 120.88	3.20	[21]
4	Strychnine	C_21_H_22_N_2_O_2_	335.1754 [M+H]^+^, 222.04, 234.09, 264.10	3.66	[22]
5	Brucine	C_23_H_26_N_2_O_4_	395.19653 [M+H]^+^ 324.11, 367.17, 350.18	3.99	[22]
6	Strychnine N-oxide	C_21_H_22_N_2_O_3_	351.17031 [M+H]^+^, 334.24, 306.16	4.33	[22]
7	Vomicine	C_22_H_24_N_2_O_4_	381.18088 [M+H]^+^, 324.14, 264.11, 306.17	5.06	[22]
8	Rutin	C_27_H_30_O_16_	611.16066 [M+H]^+^, 257.24, 285.20	6.13	[18]
9	Forsythoside A	C_29_H_36_O_15_	647.19464 [M+Na]^+^, 347.17, 321.13	6.46	[18]
10	Pinoresinol-β-d-glucoside	C_26_H_32_O_11_	543.18368 [M+Na]^+^, 218.93, 289.13	7.10	[18]
11	Phillyrin	C_27_H_34_O_11_	557.19933 [M+Na]^+^, 291.25, 218.96	10.38	[18]
12	Licorice-saponin G2	C_42_H_62_O_17_	839.40597 [M+H]^+^, 487.50, 627.38	16.92	[19]
13	Senkyunolides A	C_12_H_16_O_2_	193.1223 [M+H]^+^, 193.11, 174.95, 146.96	18.59	[23]
14	Imperatorin	C_16_H_14_O_4_	271.09648	21.80	*
15	Glycycoumarin	C_21_H_20_O_6_	369.13326 [M+H]^+^, 191.11, 285.09, 148.86	21.67	[19]
16	Curcymin	C_21_H_20_O_6_	369.13326	21.67	*
17	Ligustilide	C_12_H_14_O_2_	191.10665	21.82	*
18	3-Butylphthalide	C_12_H_14_O_2_	191.10665 [M+H]^+^, 191.07, 144.92, 172.92	21.82	[23]
19	Osthole	C_15_H_16_O_3_	245.11722	23.17	*
20	Cryptotanshinone	C_19_H_20_O_3_	297.14852 [M+H]^+^, 237.04, 251.65, 279.07	25.89	[20]
21	Tanshinone I	C_18_H_12_O_3_	277.08592	26.15	*
22	Senkyunolides P	C_24_H_30_O_4_	383.22168 [M+H]^+^, 383.32, 355.25, 365.20	29.75	[23]
23	Tanshinone II A	C_19_H_18_O_3_	295.13287 [M+H]^+^, 249.13, 277.11	30.06	[20]

(B) Negative ion mode					
No.	Components	Chemical formula	ESI^−^, m/z	RT (min)	References
1	Loganic acid	C_16_H_24_O_10_	375.12857 [M−H]^−^, 213.06, 124.96, 112.86	1.25	[21]
2	Protocatechuic acid	C_7_H_6_O_4_	153.01824 [M−H]^−^, 81.08, 108.84	1.56	[24]
3	Chlorogenic acid	C_16_H_18_O_9_	353.08759 [M−H]^−^, 191.01, 173.93	2.44	[24]
4	Paeoniflorin	C_23_H_28_O_11_	479.15478	4.23	*
5	Isoorientin	C_21_H_20_O_11_	447.09219 [M−H]^−^, 327.04, 357.10, 429.20	4.76	[21]
6	Liquiritin apioside	C_26_H_30_O_13_	549.16026 [M−H]^−^, 417.22, 297.13, 255.15	6.11	[19]
7	Lithospermic acid	C_27_H_22_O_12_	537.10275 [M−H]^−^, 295.21, 493.21	7.46	[20]
8	Dicaffeoylquinic acid	C_25_H_24_O_12_	515.11840 [M−H]^−^, 190.93, 353.13	7.64	[23]
9	Rosmarinic acid	C_18_H_16_O_8_	359.07614 [M−H]^−^, 341.15, 160.93, 196.94, 179.02	8.56	[20]
10	Salvianolic acid B	C_36_H_30_O_16_	717.14260 [M−H]^−^, 519.22, 321.20	10.08	[20]
11	Quercetin	C_15_H_10_O_7_	301.03427	11.05	*
12	Oleanoic acid	C_30_H_48_O_3_	455.35197	35.31	*

* Compared with a standard. Other components were identified through comparison with m/z and mass fragmentation patterns reported in the literature

### Lipid profile of rat serum

#### Total ion current map of rat serum (Fig. 3)

##### Validation of the UPLC-Q-Exactive Orbitrap MS method

Three methods were adopted to monitor the operating errors of the experiment and the stability of the instrument: (1) use of an advanced 5-pin QC sample balance system before injecting the experiment sample. (2) monitoring the peak height of the internal standards in all samples and then calculating their relatives standard deviations (RSDs) and (3) injecting a QC sample every 7 experimental samples. Principal component analysis of all samples, including the QC samples and the variation in each substance in the QC samples were used to test the repeatability and reliability of the instrument.

To evaluate system stability and reproducibility, we used PLSDA analysis to process the data matrix of the QC samples. In the PLSDA score plots of the KOA samples, the QC samples were clustered by positive ionization and negative ionization which indicated that the stability of the LC–MS system was satisfactory throughout the analysis (Fig. 4). In addition, the RSDs of the internal standards, lyso-PE (17:1) and SM (17:0) in positive ion mode were 7.29% and 3.83%, respectively, and the RSD of lyso-PE (17:1) was 5.98% in negative ion mode.

**Fig. 3:**
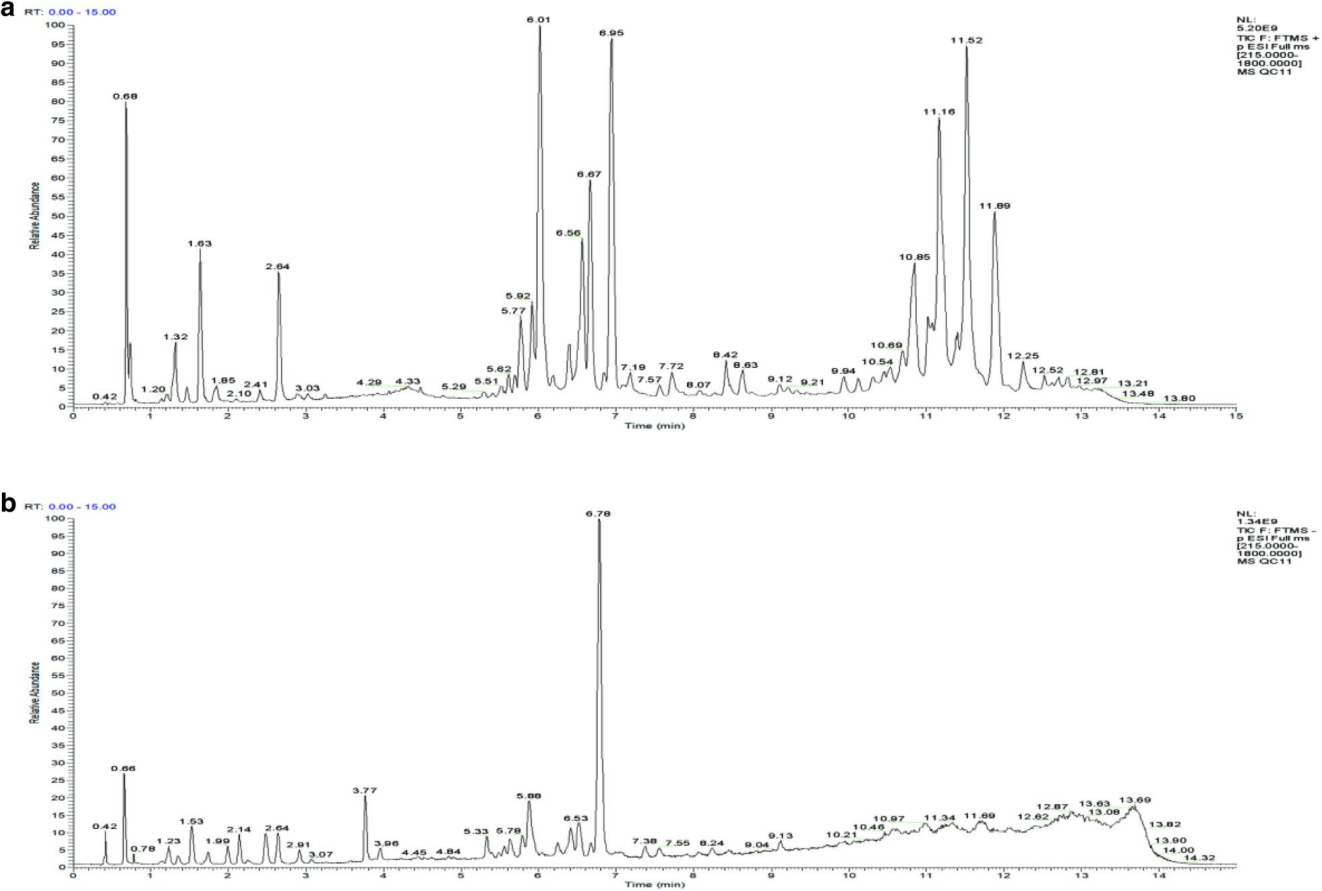
Typical total ion chromatogram (TIC) of a rat serum QC sample obtained by LC–MS in **a** positive ion mode and **b** negative ion mode

**Fig. 4:**
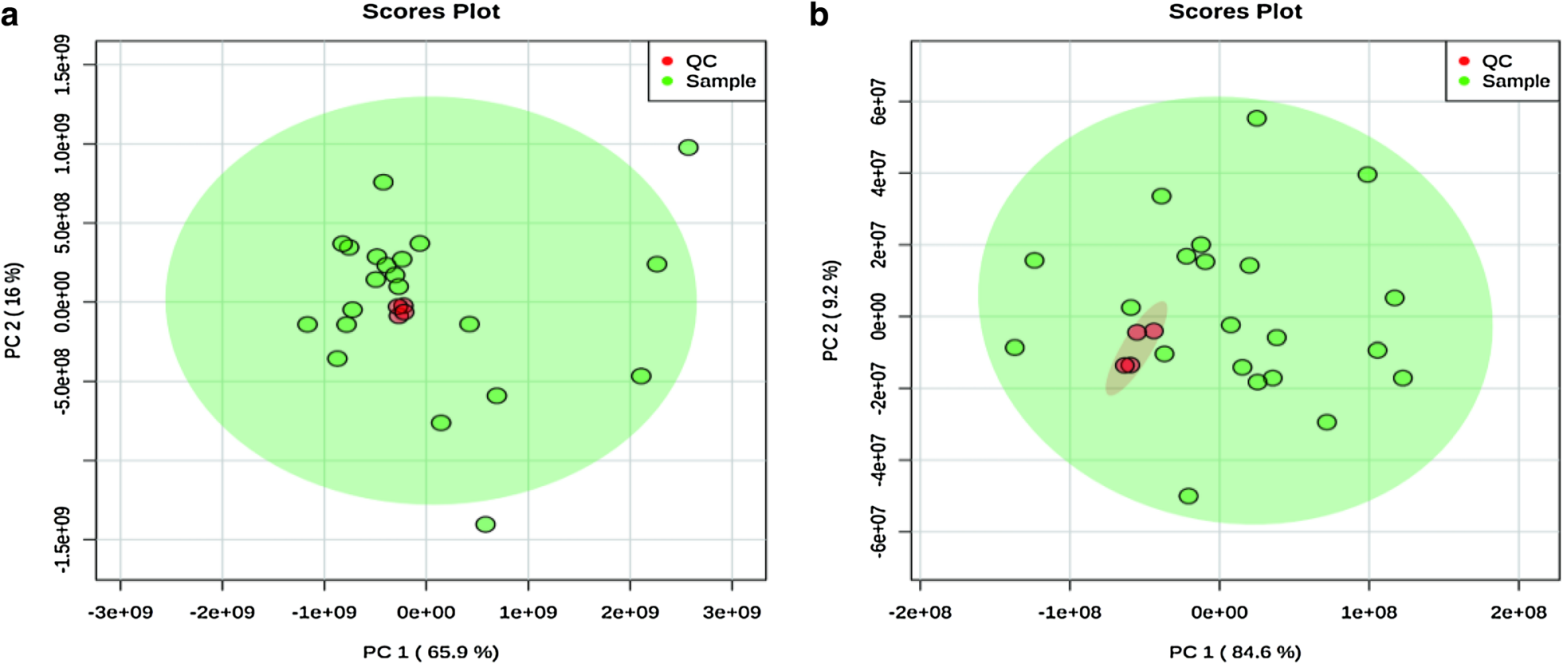
PCA of lipids in samples and QCs: **a** positive ion mode; **b** negative ion mode

##### Non-targeted lipidomics analysis

**Fig. 5:**
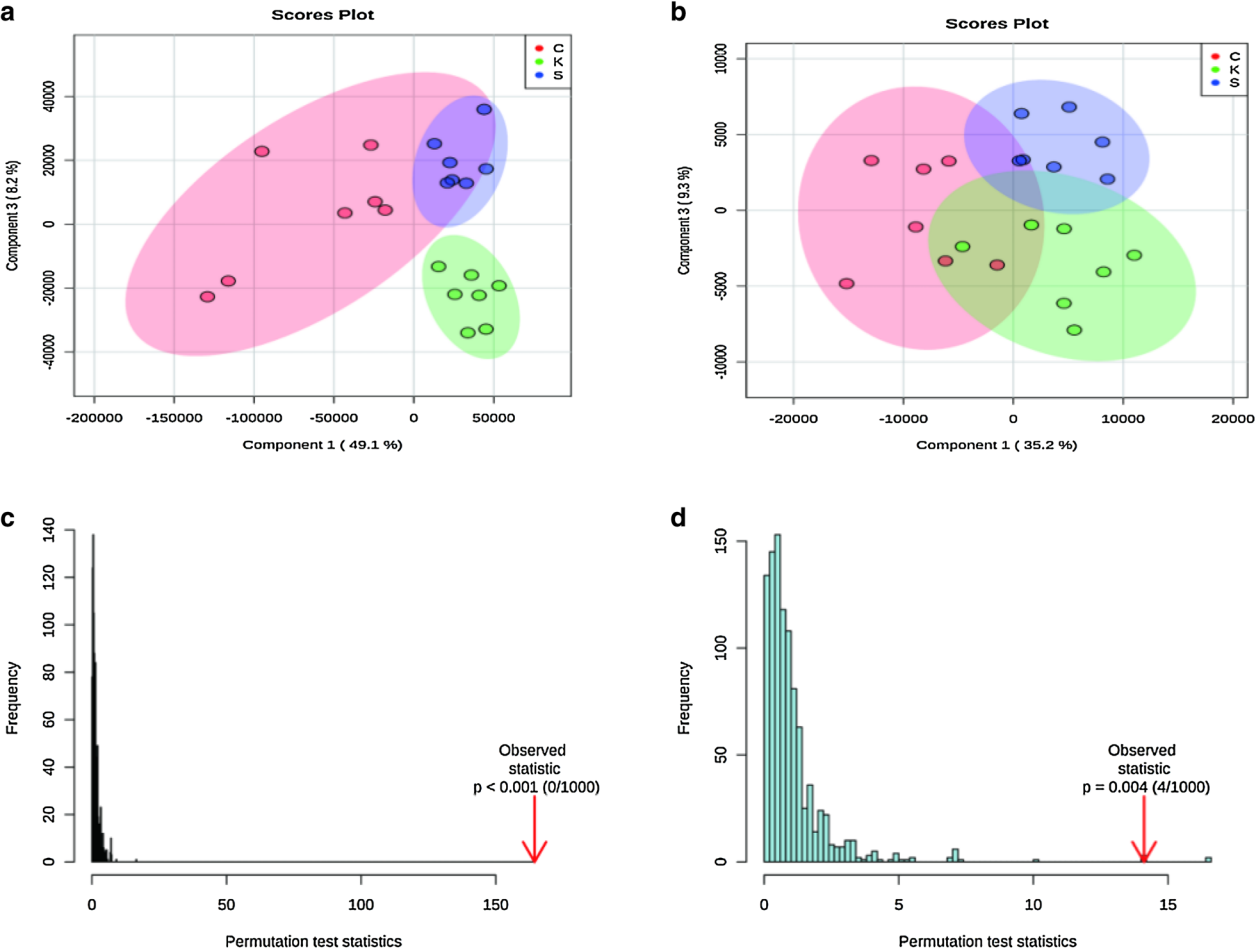
PLSDA of lipids in the KOA rats: **a**, **c** positive ion mode; **b**, **d** negative ion mode

The lipid metabolites in each group were divided according to ion modes: positive and negative. The data sets of each group for the positive and negative ion modes were analysed by PLSDA. Each point in Fig. 5 represents a sample. As shown in the figure, the control and the KOA groups showed significant separation, and the “Sanse powder” had a certain restorative effect; these effects were particularly evident in positive ion mode. The permutation test indicated that the two PLSDA models were not over-fitted, indicating that the model is valid.

##### Analysis of lipid metabolites

**Fig. 6:**
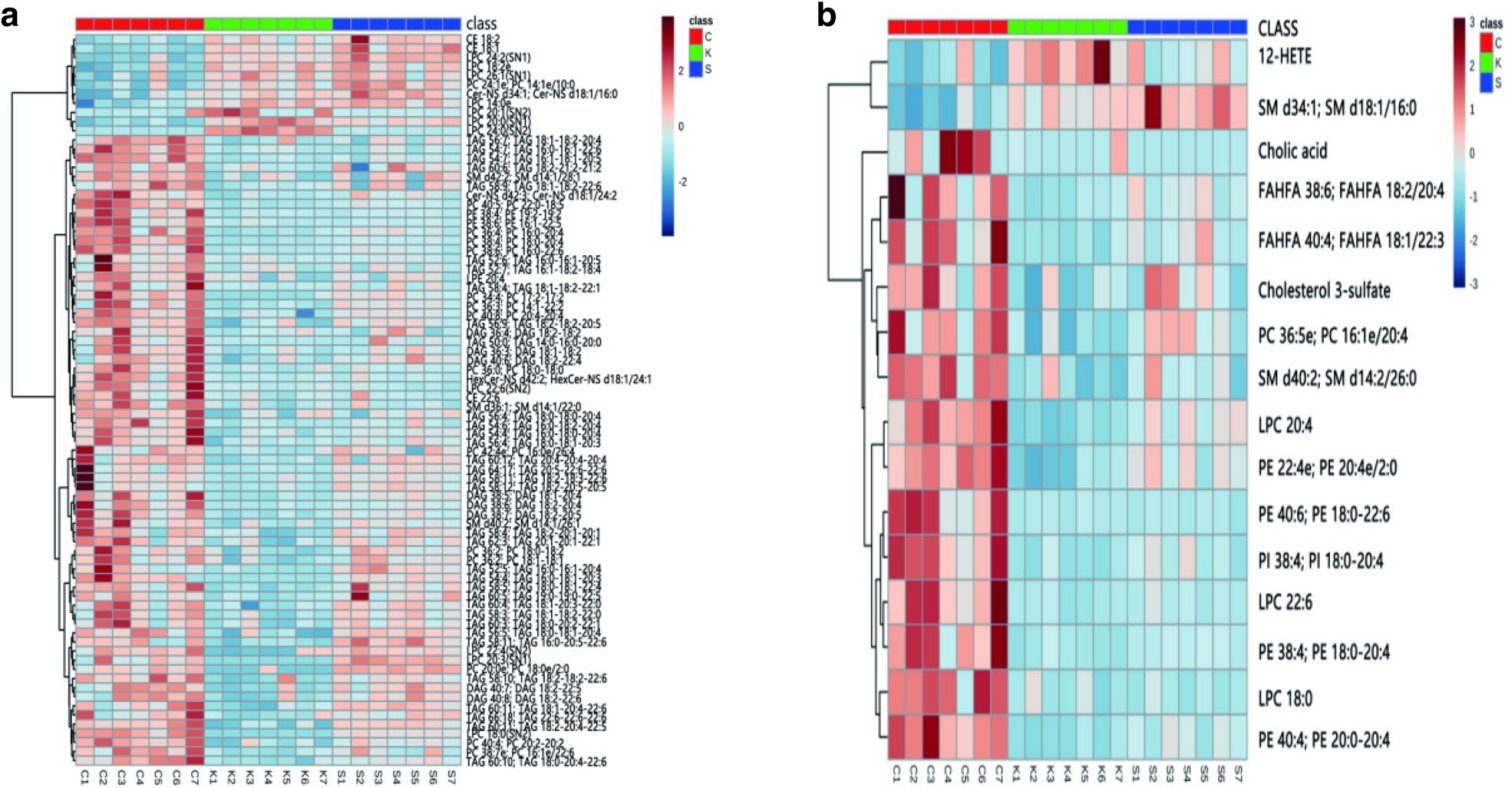
Heat map of identified differential lipids based on the positive (**a**) and negative (**b**) ion modes between the control group and the KOA group

The KW-test and FDR calibration were used to identify the lipids in each group. *P* < 0.05 was considered to indicate significance for the differences in lipids between the control and KOA groups. And significance along with an FC > 1.5 denoted differential metabolites. Heat maps were used to showed the differential metabolites between the control and KOA groups (Fig. 6). Eighty metabolites in positive ion mode and 16 metabolites in negative ion mode were determined, but not all of these differential metabolites changed in the “Sanse powder” group. Therefore, we examined the differences in metabolites between the “Sanse powder” group and the KOA group by the same method. Thirty-four metabolites in positive ion mode and 2 metabolites in negative ion mode were found to differ between the groups, among which, 20 metabolites in positive ion mode and 2 metabolites in negative ion mode were also differentially expressed between the control and KOA groups. The restored lipids and the *P* values of the FCs are shown in Table 1.

## Discussion

Lipid metabolism disorders can promote the occurrence and progression of OA via the effects of lipids on the degeneration of articular cartilage, synovitis, osteophyte formation, bone marrow oedema, metabolism and the fluidity of cell membrane components [25]. Attaining a deeper understanding of the relationship between lipid composition and KOA can aid the identification of new targets for the treatment of diseases. Lipidomics is a rapidly evolving tool that explores the potential lipid biomarkers in diseases and the biological functions of lipids in various life activities by comparing the changes in lipid metabolism networks among different physiological conditions [26].

In our study, we observed that the prominent changes in KOA rats in TAGs, DAG, PC, LPC, PE, and FAHFAs, with the greatest change observed in TAGs. Most scholars consider KOA a systemic disease, and many studies have shown that there is a certain correlation between metabolic syndrome and OA, hypertension and that glucose and lipid metabolism disorders can promote the development of OA [27, 28]. Many studies have shown a positive correlations between both TAGs and DAG and KOA [29]: this finding was slightly unique, and that study contradicted this finding.

One major phospholipid component is PC, which plays a key role in maintaining physiological functions and normal metabolism of the body. PC is found in the brain, nervous system, liver, heart, kidneys, blood and other tissues and organs and is an important component of biofilm. PC participates in cell transport, oxidative phosphorylation, phagocytolysis, and chemical and electrical excitation. In addition, PC is an important component of synovial fluid and cartilage and can inhibit cartilage hydration, scavenge oxygen free radicals and resist adhesion [30]. Several clinical studies and experiments have demonstrated that PC can alleviate the consequences of inflammation in different organs [31–33]. In general, PC can (1) reduce the interfacial tension and friction coefficient of articular cartilage, improve the physiological function of slide fluid, improve lubrication, and reduce cartilage wear; (2) reduce synovitis by controlling the synthesis and release of inflammatory mediators, such as IL-1β and TNF-α; and (3) inhibit the destruction of cartilage by inflammatory mediators in inflammatory joints, stimulate the synthesis of proteoglycan (PG), control the release of chondroitin-degrading enzymes, and accelerate the synthesis and metabolism of cartilage to stabilize and repair articular cartilage (Table 2).

**Table 2:** Significantly changed metabolites in each group

Name	FC (KOA/control)	P. adjusted (KOA/control)	FC (Sanse powder/KOA)	P. adjusted (Sanse powder/KOA)
(A) Positive ion mode				
DAG 38:5; DAG 18:1-20:4	0.24397	0.00688	2.85705	0.00820
DAG 38:6; DAG 18:2-20:4	0.05126	0.00688	5.36171	0.01939
DAG 38:7; DAG 18:2-20:5	0.26403	0.00688	2.18564	0.02927
LPC 18:0(SN2)	0.08403	0.00688	6.48339	0.00820
LPC 20:3(SN1)	0.04763	0.00688	35.57942	0.00820
LPC 24:0(SN2)	10.12183	0.00688	0.15465	0.00820
PC 20:0e; PC 18:0e/2:0	0.01511	0.02103	84.63364	0.00820
PC 34:4; PC 17:2-17:2	0.10140	0.00688	5.27038	0.01939
PC 38:4; PC 18:0-20:4	0.20891	0.00688	1.54615	0.00820
TAG 52:5; TAG 16:0-16:1-20:4	0.03330	0.02103	35.65847	0.01669
TAG 54:4; TAG 16:0-18:0-20:4	0.04690	0.00688	13.83490	0.00820
TAG 56:5; TAG 18:0-18:1-20:4	0.28945	0.02103	2.73175	0.04829
TAG 58:11; TAG 16:0-20:5-22:6	0.46693	0.02103	2.37085	0.00820
TAG 58:12; TAG 18:2-20:5-20:5	0.53100	0.01641	2.04412	0.01422
TAG 58:3; TAG 18:1-18:2-22:0	0.62809	0.03199	1.52464	0.02927
TAG 60:10; TAG 18:0-20:4-22:6	0.29493	0.00688	1.88914	0.01939
TAG 60:11; TAG 18:1-20:4-22:6	0.66509	0.02103	1.53073	0.00820
TAG 60:11; TAG 18:2-20:4-22:5	0.07639	0.00688	12.63757	0.00820
TAG 60:4; TAG 18:1-20:3-22:0	0.56576	0.02103	1.64774	0.00820
TAG 64:17; TAG 20:5-22:6-22:6	0.02183	0.03199	36.57651	0.00820
(B) Negative ion mode				
FAHFA 40:4; FAHFA 18:1/22:3	0.13704	0.00869	2.15629	0.04779
PE 40:4; PE 20:0-20:4	0.34231	0.00683	1.56335	0.04779

**Fig. 7:**
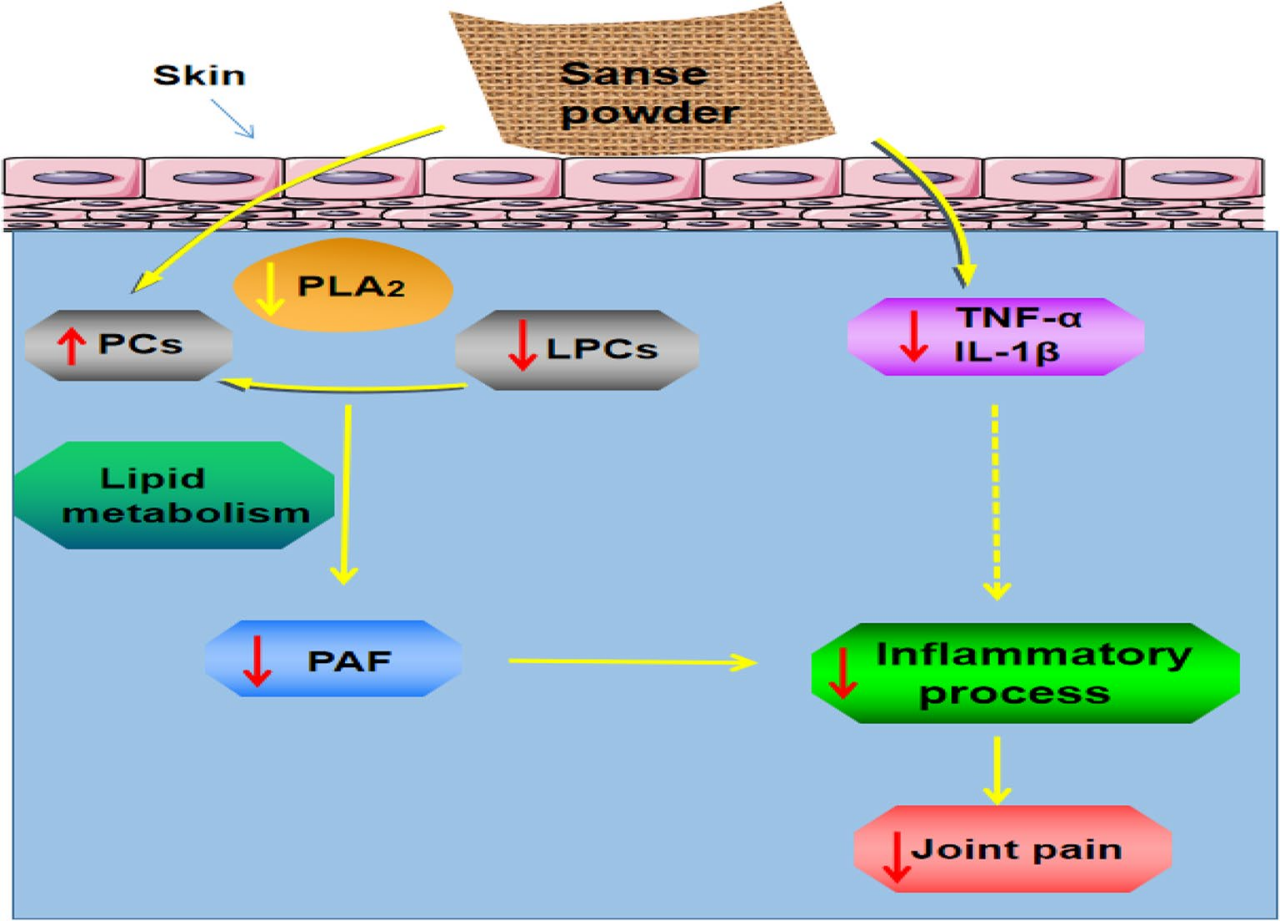
The effects of “Sanse powder” on lipid levels in KOA. *PAF* platelet activating factor, *PLA2* phospholipase A2

LPCs also play an important role in inflammatory reactions [34], some studies suggest that the ratio of LPCs to PCs is closely related to KOA [35, 36]. Furthermore phospholipase A2 (PLA2) play a key role in this process [37]. In addition, PE, a type of phospholipid (PL), is an important component of articular cartilage [38]. In our study, we found that the “Sanse powder” could restore PE levels. In addition, the concentration of FAHFAs decreased in the KOA group, which suggests that FAHFAs may play key roles in the knee joint. Therefore, the mechanisms underlying the effects of “Sanse powder” treatment on KOA may involve lipid metabolism disorders (Fig. 7).

## Conclusions

In summary, our study confirmed that “Sanse powder” had pronounced protective effects against KOA in rats. “Sanse powder” can not only reduce synovitis but also improve the cold pain threshold and MWT. Additionally, the lipidomics results showed that the levels of TAGs, DAG, PC, LPC, FAHFAs and PE on KOA rats treated with “Sanse powder” were restored to almost normal levels.

The identification and analysis of lipids led us to hypothesized that lipid metabolism disorders occurred in the KOA rats and indicated that lipids play important roles in KOA. However, our study has limitations. The experiment was a non-target experiment, so although relative quantifications of the substances were obtained, the absolute levels of the analytes were not quantified. Analyses of inflammation-related lipids in biological samples are necessary to explore their roles in cellular functioning and pathophysiological events.

## Supplementary information

**Additional file 1.** Animal ethics approval copy.

## Data Availability

All data included in this article are available from the corresponding author upon request.

## Abbreviations

KOA: knee osteoarthritis
MWT: mechanical withdrawal thresholds
H&E: haematoxylin and eosin
IL-1β: interleukin-1β
TNF-α: tumour necrosis factor-α
RT-qPCR: quantitative reverse transcription polymerase chain reaction
WB: western blot
UPLC-Q-Exactive Orbitrap MS: ultra-performance liquid chromatography combined with quadrupole-Exactive Orbitrap mass spectrometry
DAG: diacylglycerol
TAGs: triacylglycerols
LPC: lysophosphatidylcholine
PC: phosphatidylcholine
FAHFAs: fatty acid esters of hydroxy fatty acids
PE: phosphatidylethanolamine
NSAIDs: non-steroidal anti-inflammatory drugs
COX-2: cyclooxygenase 2
PGE2: prostaglandin E2
IMT: imperatorin
LC–MS: liquid chromatography–tandem mass spectrometry
CMMS: Chinese material medica
ACLT: anterior cruciate ligament transection
TIC: total ion chromatogram
RSDs: relative standard deviations
PAF: platelet activating factor
PLA2: phospholipase A2
