# Supplementary figures and images for: Interventional effects of the direct application of “Sanse powder” on knee osteoarthritis in rats as determined from lipidomics via UPLC-Q-Exactive Orbitrap MS
Source: Chin Med. 2020 Jan 23;15:9. doi: 10.1186/s13020-020-0290-5 (PMC6979340; doi:10.1186/s13020-020-0290-5)

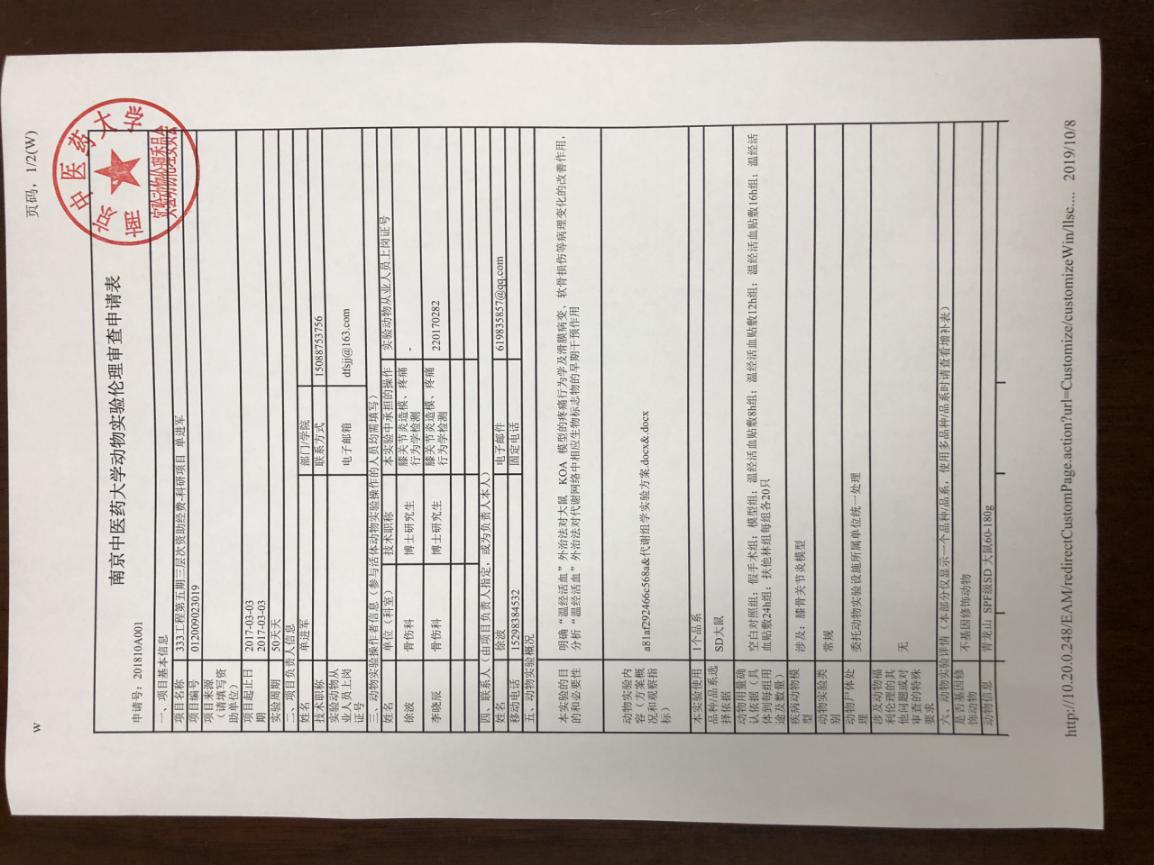


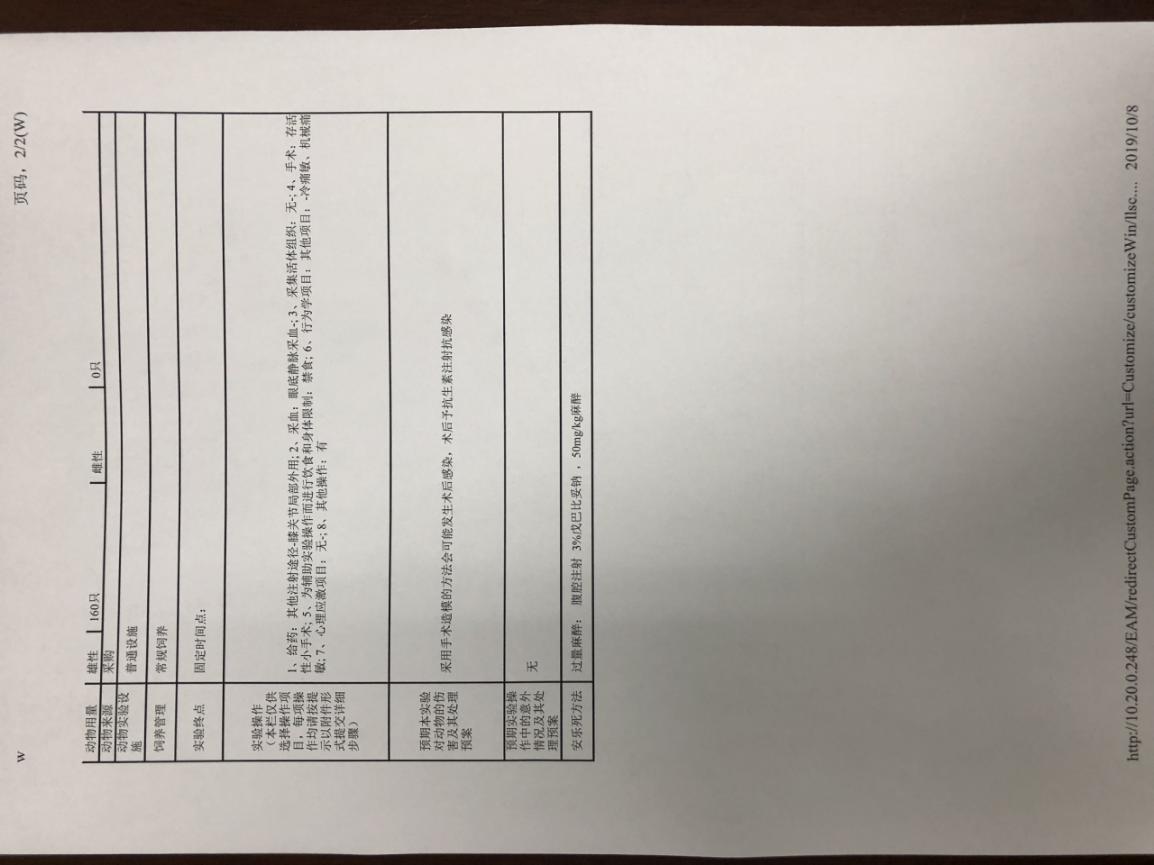

Supplement: Supplementary file 1 — Additional file 1. Animal ethics approval copy. [file 13020_2020_290_MOESM1_ESM.docx]
